# Focusing on the Emerging Role of Kainate Receptors in the Dorsal Cochlear Nucleus (DCN) and Cerebellum

**DOI:** 10.3390/ijms24021718

**Published:** 2023-01-15

**Authors:** Qin-Wei Wu, Zheng-Quan Tang

**Affiliations:** 1 School of Life Sciences, Anhui University, Hefei 230601, China; 2 Key Laboratory of Human Microenvironment and Precision Medicine of Anhui Higher Education Institutes, Anhui University, Hefei 230601, China

**Keywords:** KAR, DCN, kainate receptors, dorsal cochlear nucleus, cerebellum, cerebellum-like structure, Purkinje cells, cartwheel cells, fusiform cells, granule cells

## Abstract

Mammals have a dorsal cochlear nucleus (DCN), which is thought to be a cerebellum-like structure with similar features in terms of structure and microcircuitry to the cerebellum. Both the DCN and cerebellum perform their functions depending on synaptic and neuronal networks mediated by various glutamate receptors. Kainate receptors (KARs) are one class of the glutamate receptor family and are strongly expressed in the hippocampus, the cerebellum, and cerebellum-like structures. The cellular distribution and the potential role of KARs in the hippocampus have been extensively investigated. However, the cellular distribution and the potential role of KARs in cerebellum-like structures, including the DCN and cerebellum, are poorly understood. In this review, we summarize the similarity between the DCN and cerebellum at the levels of structure, circuitry, and cell type as well as the investigations referring to the expression patterns of KARs in the DCN and cerebellum according to previous studies. Recent studies on the role of KARs have shown that KARs mediate a bidirectional modulatory effect at parallel fiber (PF)–Purkinje cell (PC) synapses in the cerebellum, implying insights into their roles in cerebellum-like structures, including the DCN, that remain to be explored in the coming years.

## 1. Preface

The dorsal cochlear nucleus (DCN) and cerebellum are functional centers for auditory sense and motor control systems, respectively, that are important in achieving the ability to communicate with the outside world. Both structures are located neighboring each other, and the DCN is thought to be an evolutionarily precursor of the cerebellum in mammals [1,2,3,4,5,6,7,8]. Interestingly, they have similar three-layered structures and some of the same cell types and are thought to have similar mechanisms of circuitry [1,2,3,4,5]. Knowledge of either of their structures and circuitries would be very helpful in understanding the other. As intricate nerve networks are mediated and connected by synapses, glutamate is an important neurotransmitter that acts through glutamate receptors in the brain, including the DCN and cerebellum [9,10,11]. For example, as a classical metabolic glutaminergic receptor, mGluR1 has been well studied in both the DCN and cerebellum [12,13,14,15]. However, kainate receptors (KARs), as a class of ionotropic glutamate receptors, and their role in the DCN and cerebellum are far from being fully explored, and studies of their distribution in these structures in earlier years were also relatively preliminary. Here, we will systemically present the similarity of the DCN and cerebellum at multiple levels, including structure, circuitry, and cell types, and review previous studies on expression and cellular distribution patterns of KARs in the DCN and cerebellum. In the absence of studies on the role of KARs in the DCN, we summarize recent studies about the role of KARs in the cerebellum, which may provide insights for future investigation on the subject of the cerebellum-like DCN.

## 2. Cerebellum-like Dorsal Cochlear Nucleus (DCN) and Cerebellum

### 2.1. Cerebellum

The cerebellum (Latin for “little brain”) is an essential component of the body’s motor control systems. Although the cerebellum only takes up around 10% of the total brain’s volume, it contains roughly 50% of all brain neurons [6,16,17,18,19]. Motor regions provide information to the cerebellum, which integrates the information in order to fine-tune motions. The regular internal wiring of the cerebellar cortex—which consists of three layers dubbed the molecular layer, the Purkinje cell layer, and the granule cell layer—is considered to play highly precise functional roles. There are many distinct types of cells in the cerebellum, but Purkinje cells, granule cells, basket cells, stellate cells, and Golgi cells are the five most significant cell types. Of these, the excitatory neurons are the granule cells, which stimulate the synapses to which they connect by releasing the neurotransmitter glutamate. All of the other four cell types are inhibitory neurons [1,2,6,16]. The major output of the cerebellar cortex is inhibitory because Purkinje cells project into the cells of the deep cerebellar nucleus. In addition, the cerebellum contains a variety of other cell types. Excitatory glutamatergic interneurons known as unipolar brush cells are mostly found in the granule cell layer. Primary sensory interneurons called Lugaro cells are located underneath the Purkinje cell layer. The somata of candelabrum cells are found between the cell bodies of Purkinje cells, and they are dispersed throughout the cerebellar folia [1,2,20].

**Figure 1:**
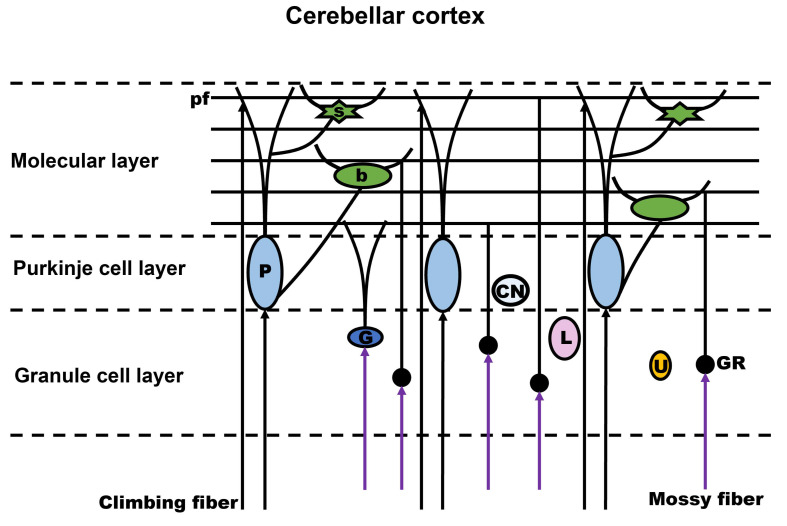
Main structure and circuitry of the cerebellum. pf, parallel fibers; P, Purkinje cell; G, Golgi cell; b, basket cell; s, stellate cell; GR, granule cell; CN, candelabrum cell; L, Lugaro cell; U, unipolar brush cell.

All of these cells have a specific location within the three-layer structure that runs throughout the cerebellum [1,2,21,22] (Figure 1). The molecular layer consists mainly of Purkinje cell dendrites, parallel fibers, and some inhibitory interneurons—mostly basket cells and stellate cells, which are dispersed between the dendritic ramifications of the Purkinje cells and the parallel fibers [1,2,21,22]. The Purkinje cell layer consists of the cell bodies of Purkinje cells, which are the output neurons of the cerebellar cortex. Purkinje cells exhibit one of the most extensively branching dendritic trees in the brain, which are a prerequisite for maintaining normal cerebellar cortex development and exercising cerebellar function [23,24]. The parallel fibers, which are descended from the axons of the granule cells, obtain the synapses from the vast dendritic trees of Purkinje cells when these dendritic trees extend the molecular cell layer. Only the Purkinje cells may emerge from the cerebellar cortex. GABA, an inhibitory neurotransmitter, is released by the Purkinje cell axon as it extends to the deep nuclei of the cerebellum. The ascending fibers, which come from the inferior olivary nucleus, also provide excitatory inputs to the dendritic trees of Purkinje cells [1,2,21,22,25]. The granule cell layer, the densest neuronal area in the cerebellum, is made up of granule cells, which are the most numerous neurons in the brain. The cerebellum receives a significant amount of excitatory input from mossy fibers, which synapse on granule cells. The granule cell sends an axon via the Purkinje cell layer to the molecular cell layer. After splitting into two lengthy fibers, this axon takes on a T shape, creating parallel fibers that connect to the dendritic spines of Purkinje cells. The Golgi cells, which are GABAergic inhibitory interneurons, connect to the granule cells via inhibitory synapses. Additionally, they receive excitatory synaptic inputs from the mossy fibers that form the cerebellar glomerular synapses [1,2,21,22,26,27]. The deep cerebellar nuclei, which project to various areas of the brain, are the output structures of the cerebellum. They can also block this signal at the subcortical level by sending a feedback projection to the inferior olive [1,2,21,22,25,26,27].

According to the above, we can know that mossy fiber and granule cells are the key components of the feedforward excitatory chain. The mossy fibers contain their terminals in the granule cell layer and stimulate granule cells and Golgi cells. They originate in the brainstem and spinal cord nuclei. They also have a collateral branch that communicates with the deep cerebellar nuclei directly by sending the same information. Granule cells are provided with both feedforward and feedback regulation by Golgi cells, which are also triggered by parallel fibers. The axons of granule cells form parallel fibers in the molecular layer and give an excitatory signal to the highly branching dendritic tree of Purkinje cells and other cells in the molecular layer. Parallel fibers also activate basket cells and stellate cells, which block excitatory inputs to Purkinje cells and play a role in the control of information integration. One single excitatory climbing fiber, originating from the cells of the inferior olive in the brainstem, innervates the dendrite of a Purkinje cell and is capable of activating it. The deep cerebellar nuclei, the cerebral cortex, the brainstem, and the spinal cord all successively receive the output of Purkinje cells. In the last stage, excitatory signals are sent out by the deep cerebellar nuclei to regulate the output of the cerebellum after Purkinje cells send out inhibitory signals and inhibit the spontaneous activity of the deep cerebellar nuclei. As a result, the inhibitory loop also involves Purkinje cells, basket cells, and stellate cells in the molecular layer as well as Golgi cells in the granule cell layer.

### 2.2. A Cerebellum-like Structure, Dorsal Cochlear Nucleus (DCN)

Cerebellum-like structures, which are histologically comparable to and presumed to share genetic ancestry with the cerebellum, have been included in the brains of all major groups of craniates except for reptiles and birds. A cerebellum-like structure termed the dorsal cochlear nucleus (DCN) can be found in almost all mammals [1,2,3,4,5]. DCN, which is located on the dorsolateral surface of the brainstem, is the first location of multisensory convergence in the auditory pathway as well as an initial site of the central auditory processing. The molecular layer, the fusiform cell layer, and the deep layer of the DCN are organized as three laminar structures similar to the layer structure of the cerebellum. Most cells with distinct morphologies and connections are in the fusiform cell layer. Glutamatergic excitatory neurons in the DCN can be divided into four classes: fusiform cells, granule cells, large cells, and unipolar brush cells. Inhibitory interneurons come in a variety of types, including Golgi cells, stellate cells, cartwheel cells, and vertical cells (also known as tuberculoventral cells) [28,29]. Although the cartwheel cell is thought to be the most similar to the cerebellar Purkinje cells, previous studies have also shown the existence of a different type of Purkinje-like cells as a class of glycinergic neurons in DCN [4,29,30,31].

**Figure 2:**
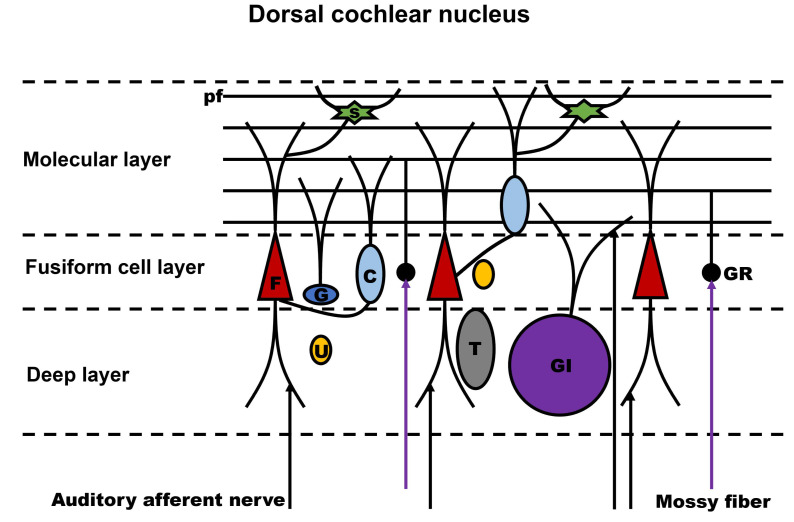
Cerebellum-like laminar structure in the DCN. Similarity of structural feature between the DCN and the cerebellum is a molecular layer that includes parallel fibers derived from granule cells. pf, parallel fibers; F, fusiform cell; G, Golgi cell; U, unipolar brush cell; s, stellate cell; C, cartwheel cell; T, tuberculoventral cell; GI, giant cell; GR, granule cell.

A schematic diagram of DCN three-laminar structure is illustrated (Figure 2). The molecular layer is mainly made up of the apical dendrites of fusiform cells, spine-covered dendrites of cartwheel cells, and parallel fibers [1,2,3,4,5,32,33,34]. The fusiform cell layer consists of the cell bodies of fusiform cells and cartwheel cells. The inferior colliculus, a relay nucleus in the auditory pathway, receives axons from the fusiform cells as the projection neurons of the DCN. Through parallel fibers that are generated from the axons of the granule cells, the fusiform cells receive auditory input on their basal dendrites and non-auditory input on their apical dendrites. Cartwheel cells, which are glycinergic and inhibit the efferent fusiform cells, are the type of cell that most resembles Purkinje cells. The cell bodies of cartwheel cells can be found near the molecular layer and fusiform cell layer boundaries in both layers, but their dendrites are mainly restricted in the molecular layer. The parallel fibers in DCN are produced by the granule cells, and they are not organized as granule cell layers in the DCN, but their function is comparable to cerebellar granule cells. The granule cells of DCN manifest as granule cell masses and mostly reside in the fusiform cell layer. Granule cells receive complex mossy fiber input from many brain nuclei, vestibular afferents, type II auditory nerve fibers, the inferior colliculus and auditory cortex [1,2,3,4,5,32,33,34]. The deep layer of DCN underneath the fusiform cell layer is the deepest and most polymorphic layer. The dendrites of fusiform cells and giant cells that receive input from the auditory nerve make up this layer. This layer also contains the cell bodies of giant cells and a large number of interneurons, e.g., Golgi cells and tuberculoventral cells. The deep layer of the DCN, which resembles the third layer of the cerebellar cortex, also contains unipolar brush cells. The layer structure, cell types, and local circuits of the DCN and cerebellum are all described above as having many similarities. Granule cells, Golgi cells, unipolar brush cells, and stellate cells, which are among the cell types, as well as the molecular layers with parallel fibers, are present in both structures [1,2,3,4,5,32,33,34,35].

**Figure 3:**
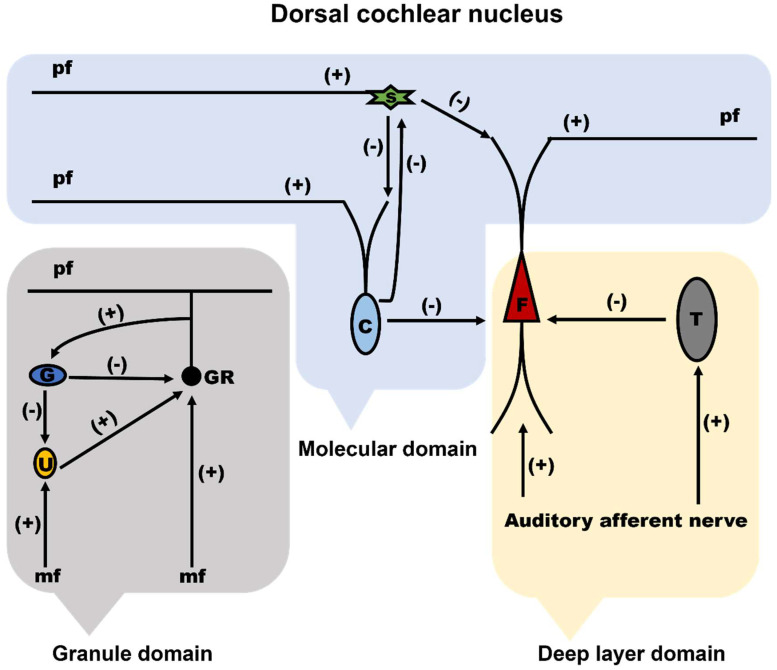
Cerebellum-like circuit domains in the DCN. Excitatory (+) and inhibitory (-) components are represented within the three functional domains of DCN, which are entitled as molecular domain, granule domain, and deep layer domain. This figure is adapted according to the published study of the ref. [36]. mf, mossy fibers; pf, parallel fibers; F, fusiform cell; G, Golgi cell; U, unipolar brush cell; s, stellate cell; C, cartwheel cell; T, tuberculoventral cell; GR, granule cell.

Although there is a molecular layer as the structural similarity between the DCN and the cerebellum, researchers have recently suggested that the DCN can be classified as three functional domains: molecular domain, granule domain, and deep layer domain [36]. In this case, an important similarity is exhibited in the DCN and cerebellum at the microcircuitry level (Figure 3). In the molecular domain of the DCN, the apical dendrites of fusiform cells, cartwheel cells, and stellate cells receive input from parallel fibers, and the activity of fusiform cells is regulated by cartwheel cells and stellate cells. Similarly, the dendrites of Purkinje cells received inputs from parallel fibers are activated by the parallel fibers, and stellate cells inhibit the excitatory inputs on Purkinje cells in the molecular layer of cerebellum. In the granule domain of the DCN, granule cells receive input from mossy fibers and are modified by unipolar brush cells and Golgi cells. In the granule cell layer of the cerebellum, cerebellar granule cells are synapsed by mossy fibers originated from brain stem and spinal cord nuclei, are provided both feedforward and feedback regulation by Golgi cells, and are modified by unipolar brush cells of cerebellum. Additionally, the basal dendrites of fusiform cells receive auditory input and are modified by tuberculoventral cells and the D-stellate cells from ventral cochlear nuclei in the deep layer domain of DCN [22,26,36,37]. It is obvious that the DCN and cerebellum have two inputs at the circuit level. The cartwheel cells and fusiform cells receive parallel fiber and auditory nerve input. Likewise, cerebellar Purkinje cells receive parallel fiber input and climbing fiber input. In both situations, the cartwheel cells, and fusiform cells of the DCN or cerebellar Purkinje cells receive a wealth of information from the first input of the parallel fibers. However, compared to DCN, the secondary input in the cerebellum is more focused. Since only one climbing fiber innervates Purkinje cells and can transmit information. Perhaps the most important distinction between the cerebellum and DCN is the existence of a climbing fiber, which is proposed to be a distinguishing trait of the cerebellum from DCN [1,3,5,7,8,38].

**Figure 4:**
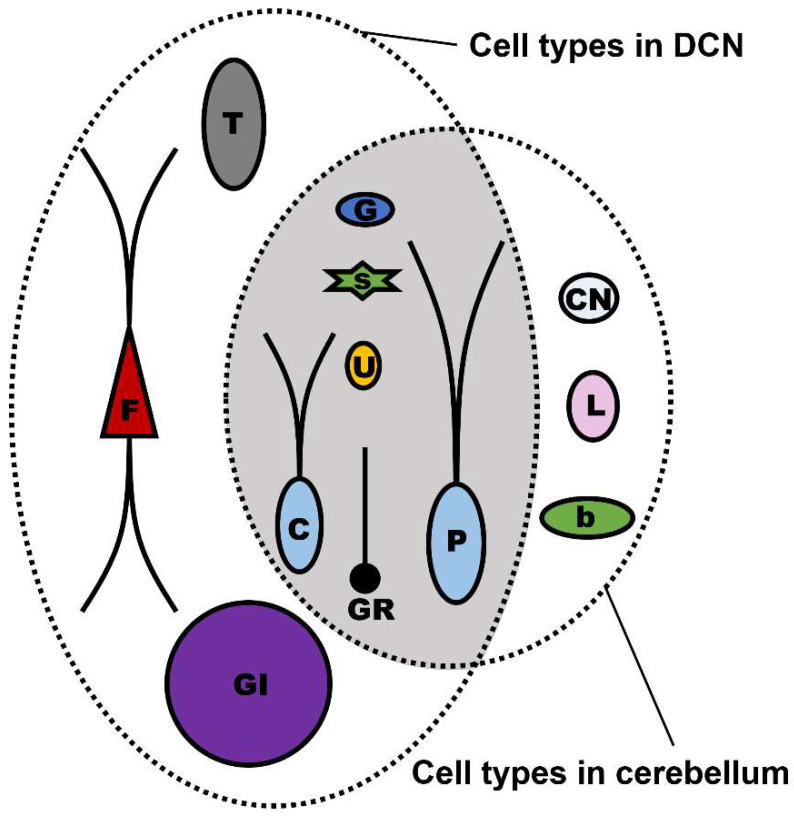
Common cell types (highlighted in the grey overlapped region) in DCN and cerebellum. The granule cells and unipolar brush cells are classified shared excitatory cell types in DCN and cerebellum. Purkinje-like cartwheel cells or Purkinje cells, Golgi cells, and stellate cells are shared inhibitory cell types. F, fusiform cell; GI, giant cell; T, tuberculoventral cell; C, cartwheel cell; G, Golgi cell; s, stellate cell; U, unipolar brush cell; GR, granule cell. P, Purkinje cell; CN, candelabrum cell; L, Lugaro cell; b, basket cell.

The supporting evidence of similar features of the DCN and cerebellum are present in the mouse models with neurological mutations, such as Purkinje cell degeneration (pcd), staggerer, and lurcher mouse models [39,40,41,42,43,44]. Cerebellar defects as a typical feature are accompanied in these mouse models. Some results have been observed in earlier investigations on these mice, indicating that the DCN might have cerebellum-like microcircuitry. Previous studies have shown that the complete population of axons in Purkinje cells of the postnatal pcd mice begins to deteriorate at two weeks. Similarly to the occurrence of abnormality of cerebellar Purkinje cells, axonal clusters produced by cartwheel cells were essentially eliminated in the pcd mice [39,40,41,42,43,44]. For the staggerer mice, few Purkinje cells could survive into adulthood because Purkinje cell dendritic growth was unsuccessful. Delayed formation of dendritic spines was observed in some surviving cartwheel cells in adult staggerer mice [39,40,41,42,43,44]. Nearly all the cerebellar Purkinje cells degenerated in the postnatal week of a mouse model known as lurcher mice. The vast majority of cartwheel cells of the lurcher mice undergo degeneration which is observed together with the degeneration of Purkinje cells [39,40,41,42,43,44]. These results are strong evidence that the genetic developmental program in both cell types may well be comparable. This comparation is further achieved in recent years, and supporting evidence is emerging at the level of cellular genetics (Figure 4). According to cell lineage studies, many cell types in the DCN and cerebellum are defined as serial sister cell types, meaning that they share same genetic developmental program. The serial sister cells from the DCN and cerebellum which originate from a type of ancestral cell are splitting descendant cells now. Several cell types from these descendant cell types are considered common cell types in both structures based not only on genetic lineage but also on phenotypic specialization [2,45,46,47]. The inhibitory neurons, including Golgi cells, stellate cells, cartwheel cells, and Purkinje cells, are serial sister cell types shared between the DCN and cerebellum. The granule cells and unipolar brush cells are classified as excitatory serial sister cell types shared by the DCN and cerebellum [2,45].

## 3. Evidence of Kainate Receptor Expression in DCN and Cerebellum

### 3.1. Kainate Receptors (KARs)

Kainate receptors (also known as kainic acid receptors) are given their name by kainic acid, a strong neurotoxin generated from the marine red alga Digenea simplex. The term “kainic” for this seaweed is taken from the Japanese name “kaininso”, which means “the ghost of the sea”. Kainate receptors (KARs) are a class of glutamate receptors, which can be split into two groups: the ionotropic and metabotropic families. The ionotropic receptors are further named mainly according to their agonist and are classified into three types: kainate (KA) receptors, N-methyl-d-aspartic acid (NMDA) receptors, and α-amino-3-hydroxy-5-methyl-4-isoxazolepropionic acid (AMPA) receptors. Although the agonists gave these receptors their names, the agonists are not completely selective. For instance, additional research has revealed that KA can also activate AMPA receptors [9,48,49,50,51,52]. AMPA receptors and KARs have analogical structures, which are cation-permeable receptor tetramers. KARs are made up of different KAR subunits. There are five types of KAR subunits, known as GluR5, GluR6, GluR7, KA1, and KA2. These subunits were subsequently given the names GluK1, GluK2, GluK3, GluK4, and GluK5 in 2009 [9,53]. GluK1, GluK2, and GluK3 compose the low-affinity subunits and can form homomeric receptors, which must be activated by high concentrations of KA or glutamate. GluK4 and GluK5 are identified as high-affinity subunits and can be activated by low concentrations of agonist, and it is believed that GluK4 and GluK5 are required for heteromerization with any of the functional receptors, including GluK1, GluK2, or GluK3 subunits [9,11,50,54,55,56].

KARs have been shown to be expressed and widely distributed throughout the mammalian nervous system. KARs are found in many cell types and interneurons of the hippocampus, cerebral cortex, dorsal root ganglia, bipolar cells of the retina, striatum, and amygdala [10,48,49,50,57,58,59,60]. Patterns of KAR expression in synapses are distinct and KARs have been identified in presynaptic and postsynaptic sites and have recently been found in the extrasynaptic sites excluded from postsynaptic compartments [61]. However, there are not many studies focusing on the expression pattern and role of KARs in the DCN and cerebellum. Here, we review the studies that have discussed expression of KARs in the DCN and cerebellum. Although these experiments with KARs present relatively preliminary data, they may provide some insight into the possible function of KARs.

### 3.2. Expression of KARs in the DCN

Since the amino acid sequences of the five KAR subunits are relatively similar and no commercial antibodies were previously available, several studies have reported the detection with KARs using distinct antibodies specifically designed to probe a particular region of amino acids expected to identify specific KARs in the DCN. The cells stained with these antibodies were observed in the different layers of the DCN and were observed as different cell types (Table 1). Although early research suggests that the subcellular localization of KARs in the DCN is at presynaptic membrane, postsynaptic membrane, and the area around the postsynaptic membrane, the exact synaptic localization of KARs in the DCN is still unknown.

An antibody (referred to as GluK2/3 antibody) was evaluated and shown to be capable of detecting GluK2 and GluK3. In the molecular layer and fusiform cell layer of the DCN, the expression of GluK2/3 staining was moderate and widespread in the DCN [62]. Another antibody (designated as GluK1-3) that can detect GluK1, GluK2, and GluK3 [63], further confirmed the previous findings of GluK2/3 antibody staining. Staining with GluK1-3 was detected in the molecular and fusiform cell layer of DCN [63,64]. These data suggest that at least GluK2 or GluK3 is expressed in the molecular layer and fusiform cell layer. GluK1 is thought to be expressed in the molecular layer and fusiform cell layer. Fusiform cells, cartwheel cells, stellate cells, granule cells, and a few neurons located in the deep layer of the DCN were stained with the GluK1-3 antibody. The staining with GluK2/3 antibody helped to partially support the results of the GluK1-3 antibody [62,63,64]. Fusiform cells, cartwheel cells, stellate cells, and several large multipolar neurons all exhibit moderate GluK2/3 antibody staining. According to some early studies on KAR transcripts, GluK2 and GluK3 are present in fusiform cells, and of them, GluK3 mRNA is also present in cartwheel cells and stellate neurons [64]. The postsynaptic area of neurons in the DCN is more strongly stained by the GluK1-3 antibody than by the GluK2/3 antibody, as shown by electron microscopy experiments. The reduced staining of the GluK2/3 antibody staining may indicate that GluK1 is expressed in the postsynaptic membrane of neurons in the DCN. Although the labeling of cytoplasm with the GluK2-3 antibody was weak, the staining of GluK1-3 was seen in the cytoplasm of DCN neurons, suggesting that GluK1 might also be expressed there. Both GluK1-3 and GluK2/3 antibodies effectively at stain synapses on the parallel fibers and spines of cartwheel cells. GluK1-3 can be detected at presynaptic terminals, although weak staining of GluK1-3 antibody occurs in parallel fibers. Similar to this result, parallel fibers show minimal staining for GluK2/3, whereas the presynaptic region of neurons shows significant positivity of staining [62,63,64]. These results also suggest that the expression pattern of GluK1 on the presynaptic membrane is similar to that of GluK2 or GluK3 in DCN.

Although there is no study on the high-affinity subunit GluK4 in DCN, a preliminary investigation on KAR transcripts has revealed that high-affinity subunit GluK5 is present in fusiform cells, cartwheel cells and some large multipolar neurons. Due to the existence of a specific GluK5 antibody, staining of GluK5 could be achieved and GluK5 was discovered to be prevalently expressed in molecular and fusiform cell layer [62]. These results are consistent with the GluK5 mRNA data. In molecular layer of DCN, GluK5 can be weakly stained in stellate cells, GluK5 demonstrated strong expression in parallel fiber. Additionally, GluK5 exhibits prevalent distribution in the cytoplasm, presynaptic and postsynaptic membranes in DCN [64,65].

### 3.3. Expression of KARs in Cerebellum

Within the cerebellum, KARs have been expressed in many cell populations, but the results were based on the studies of KAR transcripts in rodents (Table 2). GluK1 exhibited a moderate signal in the granule cell layer, and weak signal in the molecular layer by in situ hybridization [66]. At P12, Purkinje cells clearly display a strong GluK1 signal, which is maintained until adulthood. It has been noted that the immature granule cells of mice contain large amounts of GluK1 mRNA [66,67]. In rat experiments, the transcript of GluK1 was also found in a small population of granule cells [57,58].

Through development to adulthood of rodents, the parallel fibers of granule cells show the highest level of GluK2 transcript expression [67]. GluK2 signals are primarily limited to the granule cell layer in the cerebellum of adults [57,67]. In comparison to the molecular layer, GluK2 is shown more densely in the granule cell layer, and GluK2 transcripts are distributed in Golgi cells. Some studies have indicated that GluK2 is also expressed in Purkinje cells [10,57,67,68,69,70]. GluK3 transcripts are present in interneurons of the molecular layer. Throughout the postnatal period, GluK3 mRNA is exhibited in the basket cells or stellate cells of the molecular layer, and some other neurons of the granule cell layer. Some studies were shown that GluK3 are also observed in Purkinje cells [67].

For the high-affinity subunit GluK4 and GluK5, cerebellar Purkinje cells typically showed a strong signal of GluK4 mRNA expression, but the other layers of the cerebellum had low levels of signal of GluK4 [67]. GluK5 transcripts are visible in interneurons and granule cells in both the granule cell layer and the molecular layer of the cerebellum [58,62,67]. In the adult rat cerebellum, expression of GluK5 is observed in Purkinje cells, and its expression is greater than that of GluK2 and GluK3 [62,67,71].

## 4. Roles of KARs in Cerebellum Enlightening Cerebellum-like DCN

### 4.1. Role of KARs in Development of Cerebellum

KARs are substantially expressed throughout the brain and have been shown to play a role in the establishment of the neuronal synaptic network in different brain regions during the developmental stage [10]. However, studies referring to their role in cerebellar development are rare, and we therefore first briefly summarized the role of KARs in distinct regions of the developing brain, expecting to bring some insights into the possible role of KARs in cerebellar development via these recent findings. In the amygdalae, GluK1, GluK2, and GluK5 are strongly expressed during development, and endogenous KAR activity is critical for the development of the glutamatergic connections between lateral amygdala and central amygdala [72]. KARs are expressed on developing adult-born dentate granule cells and regulate their synaptic properties [73]. Low doses of KA induced high-affinity KARs activation exhibit affect spiking activity of CA3 pyramidal neurons in developing hippocampal network [74]. The intricate presynaptic and postsynaptic specializations of mossy fiber to CA3 synapses are hampered in their structural and functional development by the loss of GluK2 [10,75]. These findings support the previous studies that KAR activation enhances the maturation of mossy fiber synapses [76] and controls CA3-CA1 synapses development via the regulation of transmission at synapses [77]. The function of KARs in synapse formation and development has been investigated in cultured neurons. Overexpression of GluK1, GluK2, or GluK3 widens the active zone and boosts presynaptic puncta, whereas GluK2 or GluK5 knockdown decreases the density of presynaptic specializations [10,78]. Moreover, the role of KARs also exhibits the regulation of development of dendrites, spines and axonal filopodia. Kainate-induced activation of KARs is involved in the dendritic development in cortical neurons [79]. In a knockout mouse model of KARs, analysis of spine density revealed a significant decrease in spine density, possibly resulting in a reduction of the excitatory synapses [80]. The rapid motility of axonal filopodia is enhanced via synaptic stimulation of KARs in the developing hippocampal mossy fiber axons in order to establish synaptic contacts [81]. These results highlight the critical role KARs play in the formation of synaptic connections and demonstrate the influence KARs have on synaptic properties during neurodevelopment.

The normal development of the cerebellum depends on exact coordinated actions carried out by a large number of glutamate receptors. Some glutamate receptors, such as NMDA receptors, have been linked to climbing fiber synaptic pruning and granule cell migration [82,83,84]. The function of KARs during cerebellar development is poorly understood. However, the predominance of KAR subunit expression in the development of immature cerebellar granule cells raises the possibility that KARs play a part in synaptic transmission and maturation. KAR subunits may present varying levels of expression and different functions in the developing cerebellum [66,67,82]. Preliminary studies earlier on the KAR expression profile indicates that once an adult stage is reached, the expression of KARs containing GluK1 subunit is decreased in the granule cell layer, whereas the expression of GluK2 and GluK5 does not change [67]. According to some experimental and clinical evidence, KAR subunits have exhibited important functions during cerebellar development. GluK1 and GluK2 subunits play a role in presence of the apparent unitary conductance of kainate-type channels in developing granule cells [85], indicating the role of KAR subunits in immature granule cells at the early stage of cerebellar development. A recent case report has shown that missense variants of the *GRIK2* gene, which encodes GluK2 protein, give rise to neurodevelopmental disorder of diverse phenotype including cerebellar atrophy at an individual of about three years of age, suggesting a critical importance of KAR function during early development of the cerebellum [86]. Indeed, the predominance of GluK2 and GluK5 throughout the developmental period suggests that KAR subunits may be essential for cerebellar development [82]. Together with the distribution of KARs in the different cell types of the cerebellum as discussed in the previous paragraph, all these results imply that KARs play an important role in cerebellar development.

### 4.2. Physiological Roles of KARs in the Cerebellum

Refinement of synaptic strength during development could be a mechanism of KARs to affect the activity of synaptic network. Once development is complete and during the maturation period in the adult stage, there are other diverse mechanisms for achieving regulation, e.g., presynaptic modulation of both excitatory and inhibitory transmission and postsynaptic depolarization at excitatory synapses [87,88,89]. KARs can modulate both inhibitory and excitatory synaptic transmission via metabotropic or ionotropic signaling [9,49]. GluK2-containing KARs can control the excitability of hippocampal circuits through interaction with the modulatory cholinergic system [90]. Many studies have shown that KARs modulates GABAergic transmission when their expression is presynaptic [91]. The presynaptic KARs have been reported to mediate an increased presynaptic neurotransmitter release, giving rise to an anxiety-like behavior in mice [92]. In the adult amygdala of mice, inactivation of GluK1 has reduced GABAergic transmission to further cause an anxiety-like behavioral phenotype [93]. Postsynaptic KARs often influence inhibitory activity via their unconventional metabotropic mechanism involving G proteins and inhibition of glutamate release, which can be mediated by postsynaptic KARs in direct pathway [88,94,95]. For instance, overexpression of GluK4 promotes the efficiency of glutamate release, leading to a persistent circuit disequilibrium and influencing the outputs of amygdala [96].

**Figure 5:**
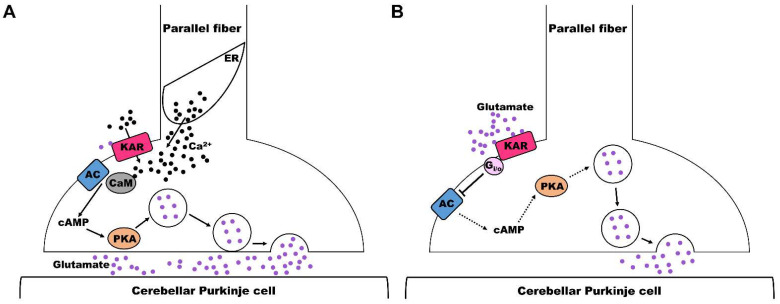
Schematic diagram of KAR-mediated facilitation or depression of glutamate release at PF–PC synapses of cerebellum. (**A**) The proposed mechanism of KAR-mediated facilitation of glutamate release at PF–PC synapses of cerebellum. Low concentration of KA enhances glutamate release through the activation of Ca^2+^ permeable KARs and the induction of Ca^2+^ from the internal reserves in endoplasmic reticulum (ER). The increase in cytosolic Ca^2+^ mediates the formation of Ca^2+^-CaM to activate AC/cAMP/PKA signaling. (**B**) The proposed mechanism of KAR-mediated depression of glutamate release at PF–PC synapses in cerebellum. High concentration of KA inhibits glutamate release via activating KARs then activating G protein and modifying AC and PKA activity.

To our knowledge, there is no study about the physiological roles of KARs in the cerebellum-like DCN structure. Considering the similarity of the DCN and cerebellum at the structural and circuitry levels, recent studies about the role of KARs in cerebellum may be helpful to understanding the potential role of KARs in cerebellum-like structures, e.g., the DCN. Most previous research suggests that KARs play a crucial role as presynaptic regulators of neurotransmitter release. This finding was initially reported in a study of the hippocampus, and this effect has been further discovered in the cortex, the amygdala, and the thalamus [97,98,99]. Electrophysiological recordings demonstrated that exogenous KAR agonists regulate transmitter release at both excitatory and inhibitory synapses in a biphasic fashion dependent upon synapse type and agonist concentration, whereas endogenous activation by released glutamate at synapses is predominantly facilitatory [11,48,49,82,100]. In agreement with what has been found in other brain regions, the bidirectional modulation of transmitter release has been recently found in the cerebellum. Activated presynaptic KARs facilitate and depress transmission at parallel fiber (PF)–Purkinje cell (PC) synapses [101,102,103]. Activation of presynaptic KARs by released glutamate at parallel fibers facilitates glutamate release to the interneurons and Purkinje cells when these fibers are subjected to a regime of low-frequency stimulation. Activation of adenylyl cyclase (AC)/cAMP/protein kinase A (PKA) signaling has recently been suggested to participate in this process (Figure 5A). In cerebellar slices, KA increased the amplitude of evoked excitatory postsynaptic currents (eEPSCs) at synapses between axon terminals of parallel fibers and Purkinje cells. KA-mediated facilitation was antagonized by the antagonist of KARs. Inhibition of PKA suppressed the effect of KA on glutamate release [103]. In contrast, with high-frequency stimulation, the synapses onto inhibitory interneurons were depressed, while synapses at Purkinje cells were still facilitated. These effects were mimicked by exogenous KAR agonists [101]. By recording evoked excitatory postsynaptic currents (eEPSCs) from cerebellar slices using the whole-cell configuration of the patch-clamp technique, high concentrations of exogenous agonist decreased the amplitude of eEPSCs and increased the number of failures at the synapses established between parallel fibers and Purkinje cells. This depression of glutamate release mediated by KAR activation was prevented by inhibition of the activation of PKA [102]. It is suggested that KARs present at these synapses mediate an inhibition of glutamate release through a mechanism that involves the activation of G-protein and protein kinase A (Figure 5B). What now seems clear is that presynaptic KARs modulate transmitter release in a bidirectional manner on cerebellum. Facilitation probably occurs through their ionotropic activity, while inhibition seems to involve noncanonical metabotropic signaling. Such differential sensitivities to the frequency of these two synapses regulate the excitation and inhibition balance of Purkinje cells and cerebellar output.

## 5. Conclusions and Perspective

Although there are few studies that have indicated the physiological role of KARs in cerebellum, the understanding of the role of KARs in the circuitry of cerebellum is far from complete. An open question is the exact role of the inhibitory and facilitatory effects of KARs on the synaptic network and the circuitry of the cerebellar cortex, which influences motor functions. Another question is whether the biphasic effects of KA on synaptic transmission at PF–PC synapses are related to the different composition of KARs, which are composed of different KAR subunits. However, our review of this subject has raised several issues of KARs which remain to be explored. First, there is a lack of precise knowledge about the cellular distribution in the cerebellum, especially of expression patterns during the developmental stage. Further studies are needed, and the role of KARs during cerebellar development must be clarified. Second, in view of the fact that both the inhibitory and facilitatory modulatory effects of KARs on glutamate release seem to require activation of PKA in the cerebellum, it is essential to identify and characterize the candidate substrates of PKA, which will help in discovering the potential signaling pathway of this action. Third, the investigation of the role of KARs may be a sign of beginning to focus on their roles in cerebellum-like structures, such as the DCN. So far, no studies on the role of KARs have been found. What is the role of KARs in the DCN, and do KARs have a similar effect of bidirectional regulation to glutamate release in the DCN? This insight may also be extended to the exploration of other cerebellum-like structures including the dorsal octavolateral nucleus, the medial octavolateral nucleus, the rostrolateral nucleus, the electrosensory lobe, and the marginal layer of the optic tectum in aquatic vertebrate groups [1]. Therefore, the directions of research on the cerebellum are also appropriate for cerebellum-like structures and must be elucidated in the future.

## Figures and Tables

**Table 1:** Expression of KAR subunits in the DCN.

DCN		GluK1	GluK2	GluK3	GluK4	GluK5	References
Molecular layer		+?	√	√	n.d.	√	[62,64]
	Stellate cells	+?	√	√	n.d.	n.d.	[64]
Fusiform cell layer		+?	√	√	n.d.	√	[62,64]
	Fusiform cells	+?	√	√	n.d.	√	[64]
	Cartwheel cells	+?	√	√	n.d.	+/-	[64]
	Granule cells	+?	√	√	n.d.	√	[62,64]
Deep layer		+/-	+/-	+/-	n.d.	n.d.	[62,64]

√, positive staining of mRNA or protein. +/-, weak or no staining. +?, uncertain expression as absence of specific antibody. n.d., no data.

**Table 2:** Transcripts of KAR subunits in the cerebellum.

Cerebellum		GluK1	GluK2	GluK3	GluK4	GluK5	References
Molecular layer		+/-	n.d.	√	+/-	√	[58,62,66]
	Stellate cells	n.d.	n.d.	√	+/-	n.d.	[67]
	Basket cells	n.d.	n.d.	√	+/-	n.d.	[67]
Purkinje cell layer		√	n.d.	√	√	√	[67,68,69,70]
	Purkinje cells	√	√	n.d.	√	n.d.	[67,68,69,70,71]
Granule cell layer		√	√	n.d.	+/-	√	[57,66]
	Granule cells	√	√	n.d.	+/-	√	[58,66,67]
	Golgi cells	n.d.	√	n.d.	+/-	n.d.	[69]

√, positive staining of mRNA or protein. +/-, weak or no staining. n.d., no data.

## Data Availability

Not applicable.
